# Analgesic Effect of the Kampo Formula Yokukansan via the Suppression of Substance P in an Experimental Rat Model of Hunner-Type Interstitial Cystitis

**DOI:** 10.7759/cureus.52238

**Published:** 2024-01-14

**Authors:** Yoshiki Tsunokawa, Mana Tsukada, Tatsuki Inoue, Masashi Tamaoka, Toshiki Mugita, Oyunchimeg Chuluunbat, Yoshiko Maeda, Takashi Fukagai, Yoshio Ogawa, Masataka Sunagawa

**Affiliations:** 1 Department of Physiology, Showa University Graduate School of Medicine, Tokyo, JPN; 2 Department of Urology, Showa University School of Medicine, Tokyo, JPN; 3 Research Administration Center, Showa University, Tokyo, JPN

**Keywords:** substance p, analgesic effect, yokukansan, kampo formula, hunner-type interstitial cystitis

## Abstract

Introduction: Yokukansan (YKS), a Kampo formula used in traditional Japanese medicine, has an analgesic effect, and is used for various pain disorders. This study investigated its analgesic effects on Hunner-type interstitial cystitis (HIC) and its mechanism of action in animal models. Methods: Rats with toll-like receptor-7 agonist (loxoribine)-induced HIC were used. Eight-week-old female Wistar rats were divided into three groups: control, HIC, and HIC-administered YKS (YKS + HIC). Bladder pain was assessed based on escape behavior using the von Frey test. Three days after HIC induction, the bladder and spinal cord were excised, and the expression of substance P (SP) was examined. Results: The pain threshold decreased significantly in the HIC group compared to that in the control group, but this decrease was suppressed by further YKS administration. The expression of SP in the bladder wall and spinal cord increased significantly in the HIC group compared to that in the control group; however, this increase was suppressed by YKS administration. Conclusion: SP is involved in the onset of bladder pain via neurokinin 1 receptors in bladder tissue. YKS may be useful for managing HIC-induced pain, and the suppression of SP secretion is one of its mechanisms of action.

## Introduction

Interstitial cystitis/bladder pain syndrome (IC/BPS) is a disorder characterized by persistent or recurrent chronic pelvic pain, pressure or discomfort perceived to be related to the urinary bladder accompanied by at least one other urinary symptom such as an urgent need to void and/or urinary frequency, diagnosed in the absence of any identifiable pathology that could explain these symptoms. Cases in which Hunner's ulcers are observed on cystoscopy are classified as Hunner-type interstitial cystitis (HIC) and distinguished from non-Hunner-type interstitial cystitis [1,2].

The underlying causes of IC/BPS are not fully understood, and the underlying mechanisms remain to be elucidated [1,2]. The involvement of vesicoureteral epithelial dysfunction, immune inflammation via activated lymphocytes and mast cells, neurogenic inflammation, malfunctioning nociceptive mechanisms, urinary toxic substances, and microbial infections have been reported [1,3]. Once bladder epithelial cells are injured by any of these causes, bladder permeability barrier is disrupted, and interstitial inflammation is induced by the influx of pro-inflammatory cytokines such as Interleukin (IL)-6 into the interstitium [4]. The inflammatory cytokines activate mast cells, leading to the release of pro-inflammatory mediators such as histamine, serotonin, and tumor necrosis factor-alpha (TNF-α). These stimulate peripheral sensory nerves and release neurotransmitters such as substance P (SP) and calcitonin gene-related peptide from peripheral nerve endings via axonal reflexes, triggering further inflammatory responses including the activation of mast cells [3,5]. Adenosine triphosphate (ATP), nitric oxide, prostaglandin, and SP produced by inflammatory reactions stimulate afferent c-fibers, resulting in pain symptoms [3,6,7]. In addition, central nerve sensitization occurs in the spinal dorsal horn, and pain symptoms and lower urinary tract symptoms are thought to become chronic [3-7]. One of the pathological conditions of central sensitization is the promotion of SP secretion from the central side of primary neurons.

In Japan, Kampo formulas (traditional Japanese herbal medicines) are also used for lower urinary tract symptoms such as pain and frequent urination [8,9]. Our clinical experience suggests that Yokukansan (YKS) [10] is effective in treating the pain symptoms of HIC. YKS is used to treat a range of pain disorders including fibromyalgia, post-herpetic neuralgia, cancer pain, phantom limb pain, headache, and trigeminal neuralgia [11,12]. Basic studies [13-17] have also suggested that it has effects. However, the effectiveness and mechanisms of action of YKS in HIC have not been firmly established. We reported that the antioxidant effect of YKS is one of the mechanisms involved in the analgesic effect of YKS on HIC pain [18]. In the present study, we focused on the excessive expression of SP in the bladder wall and spinal dorsal horn as potential causes of bladder pain [19,20], and examined the impact of YKS on SP secretion.

## Materials and methods

HIC model rats

Animals

Eight-week-old female Wistar rats were obtained from Nippon Bio-Sup. Center (Tokyo, Japan). Three animals were housed per cage (width 24 × length 40 × height 20 cm) under a 12-hour light/dark cycle in our animal facility with a controlled environment (temperature 25 ± 2°C and humidity 55 ± 5%). Food (CE-2; CLEA Japan, Inc., Tokyo, Japan) and water were provided ad libitum. All experimental procedures were approved by the Committee of Animal Care and Welfare of Showa University (certificate number: 02085; approval date: April 1, 2020). The experiments were performed in accordance with the guidelines of the Committee of Animal Care and Welfare at Showa University. Efforts were made to minimize the number of animals used and their suffering.

Groups and Induction of HIC

Eighteen rats were randomly divided into three groups: control, HIC, and YKS-treated HIC (YKS+HIC). The HIC model was constructed based on previous reports [18,21]. Briefly, pentobarbital sodium (30 mg/kg Somnopentyl; Kyoritsu Seiyaku Corporation, Tokyo, Japan) was intraperitoneally administered to the rats, which were lightly anesthetized. A quantity of 200 μL of 4.5 mM loxoribine (AG-CR1-3584; Adipogen Corp., San Diego, California, United States), a selective TLR7 agonist [18,21], was instilled transurethrally into the bladders of the rats in the HIC and YKS+HIC groups. In the control group, distilled water was used instead of loxoribine. After 60 minutes, the instilled liquid was drained.

Administration of YKS

**Figure 1:**
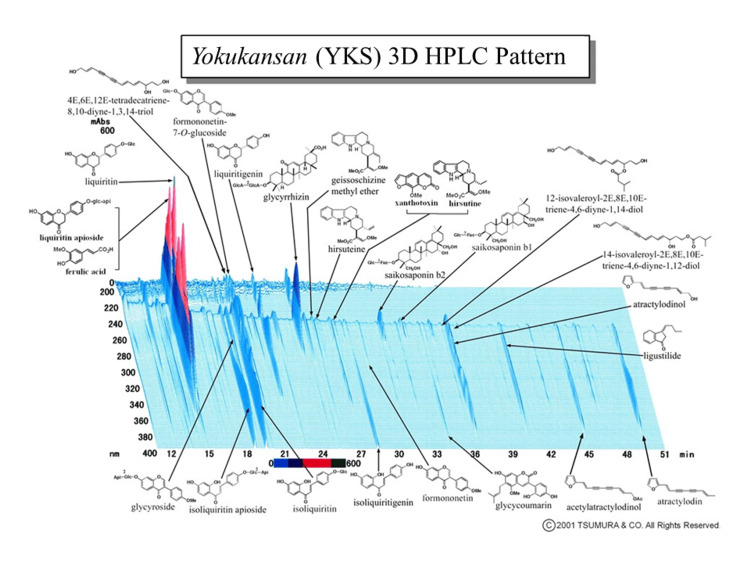
Three-dimensional HPLC profile chart of the major chemical compounds in Yokukansan (YKS) HPLC: high-performance liquid chromatography Image credit: Tsumura & Co., Japan

Dry powdered extracts of YKS (Lot No. 2110054010) were supplied by Tsumura & Co. (Tokyo, Japan). The seven crude drugs (Table 1) were mixed and extracted with purified water at 95.1°C for one hour; the soluble extract was separated from insoluble waste and concentrated by removing water under reduced pressure. A three-dimensional high-performance liquid chromatography profile chart of YKS showing the major ingredients was provided by Tsumura & Co. (Figure 1).

**Table 1:** The component galenicals of Yokukansan (YKS) Weights indicate the daily dose, and these are mixed and extracted with 600 mL of purified water (at 95.1 °C).

Official name (upper row)	Botanical family	Amount
English name (lower row)		
Rhizome of *Atractylodes lancea* De Candolle	Compositae	4.0 g
Atractylodes lancea		
Strain of *Pinus densiflora* Siebold & Zucc.	Polyporaceae	4.0 g
Poria		
Rhizome of *Cnidium officinale* Makino	Umbelliferae	3.0 g
Cnidium Rhizome		
Hook-bearing stems of *Uncaria rhynchophylla* (Miq.) Miq.	Rubiaceae	3.0 g
Uncaria Thorn		
Root of *Angelica acutiloba* (Siebold & Zucc.) Kitag.	Umbelliferae	3.0 g
Japanese Angelica		
Root of *Bupleurum falcatum* L.	Umbelliferae	2.0 g
Bupleurum		
Root of *Glycyrrhiza uralensis* Fisch.	Laguminosae	1.5 g
Glycyrrhiza		

This is an acute model, and symptoms of cystitis last for only a few days [21]. Clinically, medication is usually started after the onset of symptoms; however, in this study, YKS was pre-administered to screen for its effectiveness. YKS was mixed with powdered rodent chow (CE-2; CLEA Japan, Inc.) at a concentration of 3% and fed to the YKS+HIC group for seven days prior to the induction of cystitis. This dose was selected based on the effective doses of YKS used in our previous study [18]. Rats that were not treated with YKS were fed powdered chow only.

Measuring pain threshold

The von Frey test was performed to estimate bladder pain at baseline, before HIC induction (day 1), and on days 2 and 3. The rats were placed in a test cage with a wire mesh bottom for 30 minutes prior to testing. The response of rats to von Frey filaments of various diameters was evaluated to determine the threshold for nociceptive stimulation of the target body part [18]. The surface of the lower abdomen close to the bladder was pressed and the minimum pressure that caused retraction behavior was determined as the threshold value.

Measuring substance P

Sample Extraction

On day 3, the bladders and spinal cords of all rats were removed under deep anesthesia with sodium pentobarbital (50 mg/kg, Somnopentyl). Part of each bladder was immersed in five volumes (v/w) of protein extraction reagent (N-PER™ Neuronal Protein Extraction Reagent; Thermo Fisher Scientific Inc., Waltham, Massachusetts, United States) and then homogenized twice at 1,500 rpm for 120 seconds in a tissue homogenizer (BMS-M10N21; Bio Medical Science Inc., Tokyo, Japan). The homogenate was centrifuged at 2,000 × g for 10 minutes, and the supernatant was collected. Samples were subsequently stored at -80°C until the analysis. The remainder of each bladder and spinal cord (L6) were fixed in 4% formalin, embedded, frozen in Tissue-Tek optimum cutting temperature (OTC) compound (Tissue-Tek OCT; Sakura Finetek USA, Inc., Torrance, California, United States), and stored at -80°C until use.

Detection of SP

SP was detected using enzyme-linked immunosorbent assay and fluorescent immunostaining. SP concentrations in homogenate samples were measured using a kit (ADI-900-018; Enzo Life Sciences, Inc., Farmingdale, New York, United States). All measurements were performed according to the manufacturer’s instructions. The Pierce™ BCA Protein Assay Kit (Thermo Fisher Scientific Inc.) was used to determine the total protein concentration in samples. The SP concentrations were standardized according to the amount of protein.

The frozen bladder and spinal cord in the OTC compound were cut into 20-µm sections using a cryostat (CM1860; Leica Biosystems, Wetzlar, Germany). Sections were incubated overnight at 4°C with guinea pig anti-SP (1:500, ab106291; Abcam plc, Cambridge, United Kingdom) and rabbit anti-cytokeratin 17 (CK17) (1:1000, ab53707, Abcam plc). Sections were then incubated for two hours with fluorophore-tagged secondary antibodies: goat anti-guinea pig Alexa Fluor 555 (1:1000, A-21435; Thermo Fisher Scientific Inc.) for SP and donkey anti-rabbit Alexa Fluor 488 (1:1000, A-21206; Thermo Fisher Scientific Inc.) for CK17. Nuclei were counterstained with DAPI (4’,6-diamidino-2-phenylindole, 1:1000; Thermo Fisher Scientific Inc.). Samples were imaged using a confocal laser scanning fluorescence microscope (FV1000D; Olympus Corporation, Tokyo, Japan). The optical densities of the immunoreactive staining were measured using an appropriate software program (FV10-AW; Olympus Corporation). All values were reported as the average of five micrographs per rat. Background immunofluorescence was measured in the absence of primary antibody.

Statistical analysis

All experimental data are expressed as mean ± standard deviation. All data analyses were performed using IBM SPSS Statistics for Windows, Version 25.0 (Released 2017; Armonk, New York, United States). The statistical significance of differences between groups was evaluated using one-way analysis of variance, with post-hoc comparisons between the groups performed using Tukey's test. Statistical significance was set at p < 0.05.

## Results

Pain threshold

**Figure 2:**
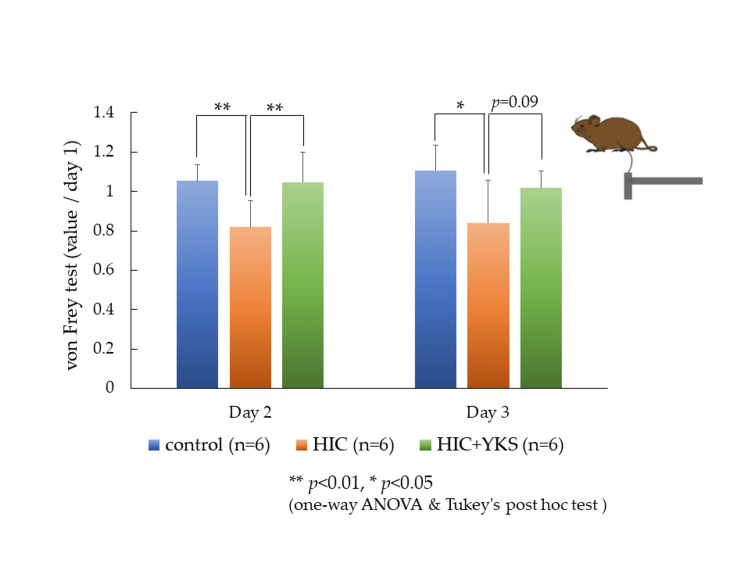
von Frey Test The values obtained on day 2 and day 3 are presented as the rate of change relative to the baseline value of 1. Significant differences: * p < 0.05, ** p < 0.01 (one-way ANOVA and Tukey's post hoc test). ANOVA: analysis of variance; HIC: Hunner-type interstitial cystitis; YKS: Yokukansan

Withdrawal thresholds were measured at baseline (day 1) and on days 2 and 3. Because the baseline values were different for each animal, the values obtained on days 2 and 3 are presented as the rate of change relative to the baseline value of 1 (Figure 2). On days 2 and 3, significant reductions in threshold values were observed in the HIC group compared to the control group, but these reductions were prevented by treatment with YKS (day 2, p = 0.01; day 3, p = 0.09).

SP in the bladder wall

**Figure 3:**
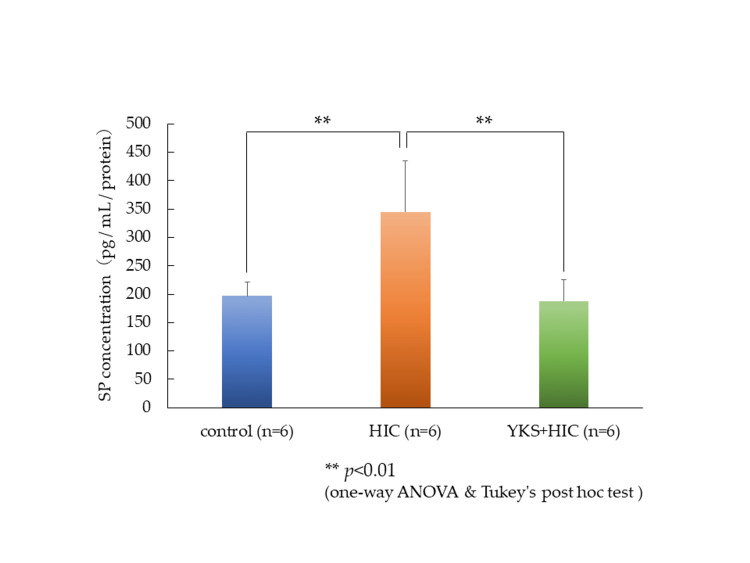
Substance P in the bladder wall (ELISA) Values are corrected by the amount of protein. Significant differences: ** p < 0.01) (one-way ANOVA and Tukey's post hoc test). ANOVA: analysis of variance; HIC: Hunner-type interstitial cystitis; SP: substance P; YKS: Yokukansan; ELISA: enzyme-linked immunosorbent assay

The expression of SP in the bladder wall was measured by enzyme-linked immunosorbent assay (ELISA), and the level in the HIC group had significantly increased compared to that in the control group; however, this increase was significantly suppressed by YKS treatment (Figure 3).

**Figure 4:**
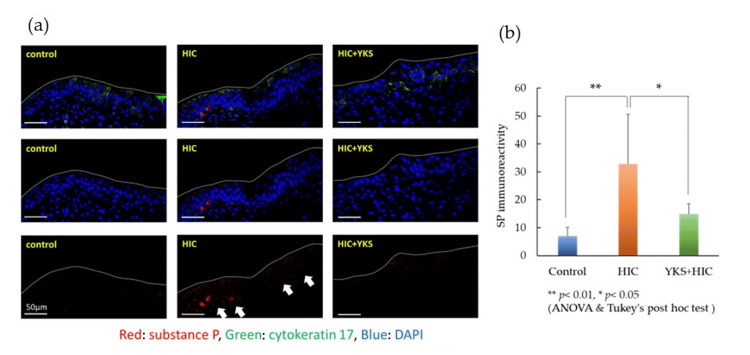
(a) Substance P (white arrows) in the bladder wall (immunostaining); (b) Immunoreactivity of SP Significant difference: ** p < 0.01, * p < 0.05 (one-way ANOVA and Tukey's post hoc test). The scale bar is 50 μm. ANOVA: analysis of variance; DAPI: 4’,6-diamidino-2-phenylindole; HIC: Hunner-type interstitial cystitis; SP: substance P; YKS: Yokukansan.

Next, SP expression was detected by fluorescence immunostaining. Red indicates SP and green indicates epithelial cells with CK17. Blue indicates nuclei. The expression of SP was elevated mainly in the connective tissue beneath the mucous membrane in the HIC group but was suppressed in the YKS+HIC group (Figure 4a). The immunoreactivity of SP was calculated (Figure 4b) and was similar to the ELISA results.

Substance P in the spinal cord

**Figure 5:**
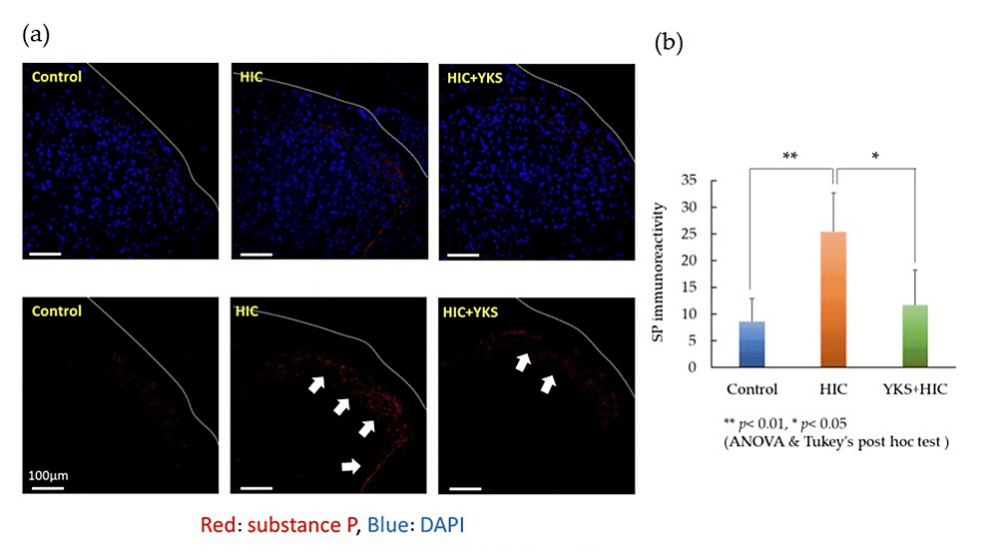
(a) Substance P (white arrows) in the spinal cord (immunostaining); (b) Immunoreactivity of SP Significant difference: ** p< 0.01, * p < 0.05 (one-way ANOVA and Tukey's post hoc test). The scale bar is 100 μm. ANOVA: analysis of variance; DAPI: 4’,6-diamidino-2-phenylindole; HIC: Hunner-type interstitial cystitis; SP: substance P; YKS: Yokukansan

The expression of SP in the spinal cord was detected using fluorescent immunostaining. Red indicates SP and blue indicates nuclei (Figure 5a). SP immunoreactivity was also calculated (Figure 5b). SP expression was significantly increased in laminae I-II of the spinal cord in the HIC group, whereas this increase was significantly suppressed in the YKS+HIC group.

## Discussion

The treatment of HIC is challenging because of the absence of a universally effective method. The treatment options vary depending on the patient’s symptoms. The pharmaceutical approaches outlined in the American Urological Association (AUA) guidelines include drugs such as amitriptyline, cimetidine, hydroxyzine, pentosan polysulfate, and cyclosporine A. Additionally, intravesical treatments such as dimethyl sulfoxide, heparin, and/or lidocaine may be used in certain instances [2].

In Japan, Kampo formulas are among the options, and medicines such as Goshajinkigan, Hachimijiogan, Choreito, and Seishinrenshiin are commonly used for urinary disorders and bladder pain [8]. Although YKS is used for various painful diseases [11,12,17], its effectiveness and mechanism of action for bladder pain associated with HIC have not been established. Pre-administration of YKS significantly inhibited the decrease in the avoidance threshold on the second day of HIC induction and showed a trend of suppression on the third day (Figure 2). Clinically, medication is usually initiated after the onset of symptoms. However, because this animal model is acute and the symptoms persist for only a few days [21], pre-administration was investigated to assess its potential efficacy in an initial experiment. In the future, we will investigate the effects of YKS in a chronic cystitis model.

YKS is a multi-component drug with multiple pharmacological actions [15-18]. Previous studies have reported its mechanisms of action, including inhibition of glutamate secretion [15], promotion of glutamate uptake by astrocytes [22], antagonism of glutamate N-methyl-D-aspartate (NMDA) receptors [23], agonistic effects on GABA_A_ and 5-HT_1A_ receptors that inhibit neurotransmission [24,25], and antioxidant properties [26]. In a previous study using the HIC model animal, we reported the involvement of the antioxidant activity [18]. In the present study, we focused on SP. Even though SP involvement has been reported in the context of cystitis manifestation and exacerbation [6,7,19,20], the effect of YKS on SP secretion remains unclear. Thus, in this study, we investigated the expression of SP in the bladder wall and spinal dorsal horn, and the influence of YKS on SP.

The HIC animal model used in this study was conceived based on the increased expression of TLR-7 in the bladder wall of human HIC patients [21]. Although changes in SP secretion have not been reported in this HIC model, it has been reported that the administration of a TLR-7 agonist to nasal epithelial cells promotes SP secretion [27]. In the HIC model, the expression of SP in the bladder wall (Figures 3, 4) and spinal dorsal horn (Figure 5) significantly increased compared to that in the control group; however, in the YKS+HIC group, this increase was significantly suppressed. These findings suggest that YKS effectively alleviates bladder pain in HIC, with one aspect of its mechanism of action involving suppression of SP secretion. To the best of our knowledge, there have been no prior reports indicating the inhibitory effect of YKS on SP secretion.

SP, a neuropeptide released by nerve endings, especially sensory nerves (C-fibers), in response to various stimuli, such as injury and inflammation, contributes to vasodilation, increased vascular permeability, and recruitment of inflammatory cells, driving the characteristic inflammation observed in cystitis. Additionally, SP influences immune cells like mast cells and leukocytes, promoting their activation and release of inflammatory mediators, further aggravating bladder inflammation. Elevated SP in the spinal cord acts as a neurotransmitter, thereby increasing pain sensitivity (hyperalgesia). They can also induce pain even in response to non-painful stimuli (allodynia), significantly contributing to persistent discomfort and pain in cystitis. SP might also be associated with heightened smooth muscle contractions in the bladder, leading to symptoms such as urinary urgency and increased frequency of cystitis. Hence, targeting SP or its receptors has become pivotal in the development of treatments to alleviate the pain and inflammation associated with this condition.

Among the components of YKS, glycyrrhizin included in *Glycyrrhiza* (Figure 1) has been reported to suppress SP levels in serum and nasal mucosa in a mouse model of allergic rhinitis [28]. Additionally, *Atractylodes Lancea* (Table 1) has been reported to inhibit the increase of SP levels in the serum of rats in a model of spleen deficiency syndrome [29]. These ingredients or crude drugs might be involved in the SP secretion inhibitory effect of YKS.

Several natural products, such as capsaicin, resiniferatoxin, and botulinum toxin (BoNT) type A, have been reported to have SP secretion suppressive effects in experiments in animal models of cystitis. BoNT primarily targets proteins within the soluble N-ethylmaleimide-sensitive factor attachment protein receptor (SNARE) complex involved in vesicle fusion and neurotransmitter release and has shown promise in reducing SP release. BoNT/A cleaves synaptosomal-associated protein-25 (SNAP-25), a key component of the SNARE complex [30]. We previously demonstrated that YKS inhibited neurotransmitter release by suppressing the overexpression of SNAP-25 [15]. This led us to speculate that YKS may share a mechanism of action like that of BoNTs. Further investigation is necessary to determine which components are active and understand their mechanisms of action. Clarifying these factors may also lead to drug development from medicinal ingredients.

One limitation of this study is its acute nature as a model, as previously mentioned. As HIC follows a chronic clinical course, we plan to further investigate YKS in chronic models to provide a more comprehensive understanding and report our findings accordingly. Furthermore, this study did not conclusively demonstrate the direct involvement of YKS in the inhibition of SP secretion. Therefore, we would be conducting future studies with the aim of clarifying the mechanism of inhibition of SP secretion, whether it involves a direct or indirect effect, and to identify its components.

## Conclusions

Kampo formulas are multi-component systems, and YKS contains many ingredients with pharmacological effects. Our previous study demonstrated the involvement of antioxidant effects in the therapeutic efficacy of YKS. The characteristics of Kampo formulas lie in multiple mechanisms acting in combination to demonstrate their therapeutic effects. It has been reported that SP is involved in the onset and exacerbation of bladder pain via neurokinin-1 receptors in the bladder tissue. YKS has the potential to alleviate pain associated with HIC, and the inhibition of SP secretion is suggested to be one of its mechanisms of action.
